# Sex Differences in Hypertension Risk: Insights from Placental Genomics and Pregnancy-Driven Vascular Programming

**DOI:** 10.3390/ijms26136034

**Published:** 2025-06-24

**Authors:** Efthalia Moustakli, Anastasios Potiris, Athanasios Zikopoulos, Despoina Mavrogianni, Nikolaos Kathopoulis, Eirini Drakaki, Ismini Anagnostaki, Ioannis Tsakiridis, Themistoklis Dagklis, Charikleia Skentou, Peter Drakakis, Panagiotis Christopoulos, Sofoklis Stavros

**Affiliations:** 1 Laboratory of Medical Genetics, Faculty of Medicine, School of Health Sciences, University of Ioannina, 451 10 Ioannina, Greece; thaleia.moustakli@gmail.com; 2 Third Department of Obstetrics and Gynecology, University General Hospital “ATTIKON”, Medical School, National and Kapodistrian University of Athens, 124 62 Athens, Greece; thanzik92@gmail.com (A.Z.); isanagnostaki3@gmail.com (I.A.); pdrakakis@med.uoa.gr (P.D.); 3 First Department of Obstetrics and Gynecology, Alexandra Hospital, Medical School, National and Kapodistrian University of Athens, 115 28 Athens, Greece; dmavrogianni@med.uoa.gr (D.M.); nickatho@gmail.com (N.K.); eirinidrak@med.uoa.gr (E.D.); 4 Third Department of Obstetrics and Gynecology, General Hospital Ippokratio, Medical School, Aristotle University of Thessaloniki, 546 42 Thessaloniki, Greece; iotsakir@gmail.com (I.T.); tdagklis@gmail.com (T.D.); 5 Department of Obstetrics and Gynecology, Medical School, University of Ioannina, 451 10 Ioannina, Greece; haraskentou@uoi.gr; 6 Second Department of Obstetrics and Gynecology, Aretaieion University Hospital, Medical School, National and Kapodistrian University of Athens, 115 28 Athens, Greece; panchrist@med.uoa.gr

**Keywords:** hypertensive disorders of pregnancy, placental genomics, sex differences, X-linked genes, epigenetics

## Abstract

The prevalence, pathogenesis, and long-term consequences of hypertension differ significantly across the sexes, and pregnancy is a special physiological stress test that can reveal a woman’s underlying cardiovascular sensitivity. In addition to being direct risks to the health of the mother and fetus, hypertensive disorders of pregnancy (HDPs), especially preeclampsia, are also reliable indicators of future hypertension and cardiovascular disease in those who are afflicted. Fetal sex has a substantial impact on maternal vascular adaptation, according to new data from placental transcriptomics and epigenetics. This may be due to variations in the expression of angiogenic, immunomodulatory, and vasoactive genes. Sex-specific patterns of placental function, inflammation, and endothelium control are specifically influenced by X-linked gene dosage, escape from X-inactivation, and sex chromosomal composition. These biological variations highlight the placenta’s potential function as a mediator and indicator of maternal cardiovascular risk, and they may help to explain why the incidence and severity of hypertensive pregnancy challenges vary depending on the fetal sex. The purpose of this review is to summarize the state of the art regarding how placental genetics and fetal sex influence maternal hypertensive risk both during and after pregnancy. Additionally, it will investigate how these findings may influence sex-specific cardiovascular screening, prediction, and prevention methods.

## 1. Introduction

Hypertension is a leading cause of cardiovascular morbidity and mortality, affecting more than 1.3 billion individuals worldwide [1]. Despite clear sex differences in its prevalence, underlying causes, and long-term consequences, hypertension is frequently approached as a single clinical entity [2]. The influence of sex hormones and vascular aging on blood pressure regulation contributes to the higher incidence of hypertension observed in men during early adulthood. In contrast, women have a considerable increase in incidence after menopause. The effectiveness of therapies for both sexes may be limited because the majority of professional recommendations and therapeutic approaches are still mostly sex-neutral [3].

Recent advances in molecular biology, vascular genomics, and developmental physiology have catalyzed interest in sex-specific mechanisms underlying cardiovascular disease [4]. A unique physiological model to study these pathways is provided by pregnancy, a time of significant hemodynamic and immunological adaptation. The placenta contributes to the extensive remodeling of the maternal circulatory system during pregnancy, facilitating adaptive changes in endothelial function, a reduction in systemic vascular resistance, and an increase in cardiac output. A healthy pregnancy depends on these vascular changes, which are closely regulated by immunological, endocrine, and epigenetic mechanisms [5].

Crucially, pregnancy complications such as preeclampsia and gestational hypertension serve as early markers of heightened long-term cardiovascular risk, particularly in women. These disorders are not only characterized by aberrant placental development and function but also reflect deeper dysregulation in vascular and immune signaling [6,7]. Pregnancy can be thought of as a physiological stress test for the maternal cardiovascular system, with the potential to uncover latent vulnerabilities and contribute to the development of future hypertension risk [8].

Recent research in placental genomics has revealed sex-specific transcriptional and epigenetic mechanisms that regulate maternal vascular adaptation and influence fetal development [9,10]. Male and female fetuses have considerably different placental gene expressions, affecting inflammation, oxidative stress (OS) pathways, and angiogenic processes. Sexually dimorphic signals can influence maternal endothelial function and lead to different cardiovascular risk profiles. Furthermore, the relationship between maternal vascular health, placental signaling, and fetal sex provides a new framework for understanding the developmental etiology of sex-specific illness [11,12,13].

This review explores how pregnancy, particularly the molecular and genetic functions of the placenta, elucidates the mechanisms underlying sex differences in hypertension risk. The concept of “Vascular Programming” refers to the mechanism by which environmental exposures during early life or gestation, such as placental signals and fetal sex, elicit persistent structural and functional changes in the maternal vascular system. These alterations may influence long-term cardiovascular outcomes and continue beyond pregnancy. To illustrate a model demonstrating how sex-specific signals during gestation exert long-term effects on maternal cardiovascular health, we integrate evidence from placental transcriptomics, vascular epigenetics, and pregnancy-induced physiological changes. Addressing these pathways highlights the necessity of including sex and reproductive history in cardiovascular risk assessment and management, while also elucidating precision medicine approaches to hypertension.

## 2. Biology of Sex Differences in Hypertension

Sex differences in hypertension stem from complex interactions among genetic, hormonal, and developmental factors. Biological sex is a fundamental factor influencing both the risk of hypertension and its clinical manifestation, despite the modifying effects of lifestyle and environmental factors on blood pressure regulation. These differences result from the interaction of sexually dimorphic placental biology, sex chromosomes, and sex steroid hormones, each of which affects vascular tone, renal function, and inflammatory responses differently in each sex [2,14].

### 2.1. Role of Sex Chromosomes

Independent of gonadal hormones, the sex chromosome complement (XX in females, XY in males) contributes to hypertension risk through mechanisms involving gene dosage and genomic imprinting [15]. Genes on the X chromosome, such as ACE2, NOX1, and ATRX, control vascular function, OS, and renin–angiotensin system (RAS) activity. Additional complexity arises from X-chromosome inactivation in females, as certain X-linked genes escape silencing, resulting in sex-specific differences in gene dosage [16]. Additionally, the Y chromosome contains regions related to blood pressure regulation, such as renal salt processing and SRY-dependent sympathetic tone modulation [17]. The genetic component of sex-biased blood pressure regulation has been highlighted using the Four Core Genotypes in mouse models, indicating that the sex chromosomal complement can affect hypertension independently of gonadal sex [18].

### 2.2. Role of Sex Hormones

Sex hormones, especially androgens and estrogens, have a major impact on immunological regulation, renal sodium management, and vascular homeostasis [19]. In general, estrogens are vasoprotective, encouraging vasodilation, the activation of endothelial nitric oxide synthase (eNOS), and a reduction in vascular inflammation. These effects are transmitted through both genomic and non-genomic pathways by estrogen receptors (ERα, ERβ, and GPER1) that are expressed in vascular, renal, and immunological organs [20,21]. Conversely, androgens such as testosterone have been associated with increased sympathetic activity, pro-inflammatory states, and vasoconstriction, particularly in elderly men [22].

Postmenopausal women experience a reduction in estrogen’s protective effects, which correlates with a heightened risk of developing hypertension. The importance of estrogen signaling in preserving blood pressure regulation is further demonstrated by findings from hormone replacement therapy and receptor deletion experiments in animal models [23]. Furthermore, while being less researched, progesterone and relaxin are crucial for vascular compliance and renal vasodilation throughout pregnancy, indicating that changes in these hormones may have long-term cardiovascular effects [24].

### 2.3. Role of Placental Biology

The placenta is a critical mediator of maternal cardiovascular adaptation during pregnancy and exhibits sexually dimorphic gene expression that impacts maternal physiology. Studies have shown that male and female placentas differ in transcriptomic profiles, methylation patterns, and mitochondrial function. These discrepancies are linked to variations in angiogenic signaling, placental efficiency, and OS resilience, all of which affect maternal vascular tone and endothelial function [25,26].

Female placentas are generally more resilient in response to stressful intrauterine environments, whereas male placentas typically show increased expression of genes related to OS and inflammation. These biological variations could explain why male fetuses have a higher risk of developing preeclampsia during pregnancy. Moreover, the placenta influences maternal immune and vascular responses by releasing hormones, cytokines, microRNAs, and extracellular vesicles that vary by fetal sex [27].

Significantly, these sex-specific placental signals affect not only the intrauterine environment but may also exert lasting effects on the mother’s cardiovascular health. Prenatal difficulties, particularly those associated with aberrant placentation, appear to be early markers of maternal risk for chronic hypertension, and fetal sex may play a role in these correlations [28].

## 3. Role of Pregnancy

Pregnancy is widely acknowledged as a natural physiological stress test, one that can reveal subclinical predispositions to cardiovascular disease in women. The substantial hemodynamic demands of gestation necessitate the precise coordination of the vascular, renal, metabolic, and immunological systems [29]. When these systems fail to adapt appropriately, HDPs—including gestational hypertension and preeclampsia—may develop. In addition to complicating immediate obstetric outcomes, these disorders serve as markers for future cardiovascular diseases, including chronic hypertension [30].

The placenta is more than simply a passive channel between the mother and the fetus; it is a highly dynamic and genetically active organ that regulates maternal physiology [31]. The placenta, derived from the fetus, has a unique genomic makeup shaped by both inherited genetic factors and environmental influences such as fetal sex and maternal health [32]. Progress in placental transcriptomics and genomics has revealed the placenta’s essential role in orchestrating endocrine signals, immune tolerance, and vascular remodeling throughout pregnancy. Maternal cardiovascular health is closely associated with these processes, both during pregnancy and long after birth [33].

### 3.1. Pregnancy as a Physiological Stress Test

A robust and growing body of epidemiological data has established a strong association between HDP and long-term maternal hypertension. Within five to ten years after giving birth, women with a history of preeclampsia are two to four times more likely to develop persistent hypertension, according to meta-analyses (Table 1). The risk remains evident despite controlling for traditional cardiovascular risk factors like age, BMI, and smoking, indicating that pregnancy complications offer important prognostic information [34].

Moreover, this risk appears to follow a dose–response relationship, with earlier-onset or more severe HDP conferring greater long-term hypertension risk. Any aberrant blood pressure regulation during pregnancy may indicate underlying vascular dysfunction or maladaptive responses, as even milder types of gestational hypertension are linked to negative cardiovascular trajectories [35].

HDP has also been linked to an increased risk of heart failure, myocardial infarction, and stroke in later life by large-scale cohort studies as the Nurses’ Health Study, the UK Biobank, and the HUNT Study [36]. Significantly, in younger women, pregnancy-related complications may provide superior prognostic information compared to traditional cardiovascular risk factors, highlighting the essential consideration of reproductive history in cardiovascular risk evaluation [37].

While the molecular mechanisms behind HDP, particularly genetic susceptibility, remain unclear, these findings have resulted in their incorporation into cardiovascular disease prevention guidelines for women [38].

### 3.2. Placentation and Maternal Cardiovascular Health

The transcriptional landscape of the placenta reflects its crucial role in modulating vascular tone, angiogenesis, and systemic inflammation (Table 2). Several studies have identified significant placental genes that are differentially expressed in pregnancies complicated by high-blood-pressure conditions, such as PE, a condition closely related to long-term maternal hypertension risk. Soluble endoglin (ENG) and Flt-1 (FLT1) are the most extensively studied anti-angiogenic factors [39]. The soluble version of VEGF receptor 1 (sFlt-1) works by sequestering circulating VEGF and PlGF, preventing them from interacting with endothelial receptors. This alters VEGF signaling, leading to endothelial dysfunction, decreased nitric oxide generation, and increased vascular resistance—hallmarks of preeclampsia which may also cause eventual maternal hypertension [40]. Overexpression of ENG and FLT1 impairs pro-angiogenic signaling, resulting in decreased endothelial function [39].

The disrupted regulation of hypoxia-related genes such as HIF1A and HIF2A impairs spiral artery remodeling and trophoblast invasion, both critical processes in the pathophysiology of hypertensive placental disorders. Crucially, these disorders frequently result in the downregulation of pro-angiogenic genes, including VEGFA and PGF, contributing to an anti-angiogenic maternal environment [41].

The aberrant placental expression of hypoxia-inducible factors, particularly HIF1A and HIF2A, indicates inadequate oxygen sensing and contributes to poor spiral artery remodeling. These transcription factors promote anti-angiogenic mediators like sFlt-1 and inflammatory cytokines while inhibiting pro-angiogenic genes like VEGFA. Sustained placental hypoxia caused by maladaptive HIF activity affects maternal systemic vascular tone. It exacerbates OS with effects that differ by fetal sex, with male pregnancies having stronger HIF stabilization under stress conditions [42].

These modifications are often paralleled in maternal circulation, with circulating levels of placental factors serving as early biomarkers of vascular dysfunction. In addition to coding genes, epigenetic processes like DNA methylation and histone modifications alter placental function in response to environmental stressors such as maternal obesity, inflammation, and psychological stress. These molecular changes influence maternal endothelial responses, systemic vascular resistance, and OS levels, highlighting their role in maternal cardiovascular adaptation and risk [43,44].

### 3.3. Fetal Sex and Sex-Specific Placental Signals

Fetal sex is an intrinsic biological variable that significantly alters placental development, function, and gene expression (Table 3). Numerous transcriptomic studies have demonstrated widespread sex-biased expression in the placenta, independent of pregnancy complications. These differences influence core placental functions such as mitochondrial metabolism, OS handling, and immune tolerance—all of which affect maternal vascular adaptation [45].

In placentas from male fetuses, there is often an upregulation of genes linked to inflammation and OS, which may amplify maternal vascular load and systemic inflammatory responses [46]. Conversely, female placentas indicate increased transcriptomic flexibility, allowing for improved adaptive responses to environmental challenges hypoxia and nutrient deficiency [47]. Non-coding RNAs that regulate trophoblast invasion, angiogenesis, and immune function—including microRNAs (such as miR-210 and miR-155) and long non-coding RNAs (like XIST)—are also affected by fetal sex. These molecules can be released into the maternal bloodstream via extracellular vesicles, where they influence vascular tone and endothelial gene expression [48].

MicroRNA expression in the placenta differs by sex, contributing to variable maternal vascular adaptations. For example, miR-210, which is increased in male placentas, has been linked to impaired mitochondrial activity and endothelial cell migration. Furthermore, miR-155, which is likewise male-biased, reduces eNOS expression and increases inflammation. These sex-specific miRNA signatures are detectable in maternal circulation and may serve as molecular mediators between fetal sex and maternal cardiovascular risk [49].

Furthermore, male fetuses have been linked with increases in maternal blood pressure, vascular resistance, and the risk of preeclampsia. These clinical findings indicate that placental signals influenced by fetal sex may contribute to modifications in maternal cardiovascular function during pregnancy. These signals may result in enduring epigenetic changes in the immune system and endothelial cells, contributing to the risk of postpartum hypertension [50].

The placenta serves as both a transient fetal organ and a predictor of maternal cardiovascular outcomes, with sex-specific gene expression that may raise the probability of hypertension long after birth. Insight into these pathways paves the way for advancements in precision medicine, early therapeutic intervention, and biomarker identification for cardiovascular disease in women [51].

**Table 3:** Sex-specific differences in placental biology and implications for maternal vascular health.

Aspect	Male Placenta	Female Placenta	Implication for Maternal Health
Inflammatory gene expression [52]	Elevated (e.g., *IL*-*6*, *TNF-α*)	Lower	Higher systemic inflammation in mother
OS markers [53]	Increased	Lower, more regulated	Endothelial dysfunction risk
Angiogenic balance [54]	More prone to sFlt-1 overexpression	More stable PGF expression	Greater anti-angiogenic signaling with male fetuses
Mitochondrial function [55]	Less efficient, higher ROS	More efficient, adaptable	Energy stress and vascular damage in mother
microRNA profiles [56]	*miR*-*210*, *miR*-*155* elevated	Distinct adaptive signatures	Sex-specific epigenetic regulation of maternal endothelium

### 3.4. Genetic and Epigenetic Mechanisms Revealed by Pregnancy

While pregnancy complications often serve as clinical red flags for future disease, they may also act as windows into a woman’s underlying genetic risk. Several studies indicate that pregnancy may reveal hidden genetic vulnerabilities linked to immunological dysregulation, endothelial activation, and vascular dysfunction—features that are highly similar to risk profiles for hypertension and cardiovascular disease [57] (Table 4).

Genome-wide association studies (GWASs) have identified shared risk loci between HDP and essential hypertension. For instance, preeclampsia and chronic hypertension are linked to variations in genes including FTO, MTHFR, eNOS (NOS3), and SH2B3. Specifically, SH2B3 encodes a protein that is involved in vascular and immunological communication and has been linked to inflammatory and hypertensive phenotypes [58,59].

Additionally, polygenic risk scores (PRSs) for hypertension are increasingly used to quantify the cumulative genetic burden. Even in the absence of previous cardiovascular disease, recent research shows that women with a high PRS for hypertension are more likely to acquire HDP, indicating that pregnancy may serve as a phenotypic “trigger” in genetically predisposed people [60].

Moreover, recent studies suggest that maternal–fetal gene interactions, particularly within placental tissue, may influence pregnancy outcomes. For example, maternal vascular reactivity variations may be increased by fetal or placental genotypes that promote anti-angiogenic factor production, such as sFlt-1 or sEng. These gene–environment interactions may help explain why certain women develop HDP in one pregnancy but not others [61,62].

Together, these findings corroborate the paradigm that pregnancy acts not as an etiological agent of hypertension but rather as a physiological stressor that reveals an individual’s latent predisposition, encompassing genetic, molecular, and physiological factors [63].

**Table 4:** Genetic and molecular factors linking pregnancy complications to long-term hypertension.

Gene/Locus	Function	Associated with	Implication
SH2B3 [64]	Immune and vascular regulation	PE, HTN	Shared inflammatory and hypertensive pathways
FTO [64]	Metabolic and vascular signaling	Obesity, HDP, HTN	Metabolic–vascular interface
eNOS (NOS3) [65]	NO production, vasodilation	HDP, endothelial dysfunction	Impaired vascular tone and endothelial function
MTHFR [66]	Methylation, homocysteine metabolism	PE, HTN	Endothelial stress, oxidative damage
Hypertension PRS [67]	Cumulative genetic burden	HDP, later-life HTN	Predictive of postpartum risk

## 4. Implications for Precision Medicine

The increasing evidence that sex-related disparities in hypertension result from the interplay of genetic, hormonal, and placental pathways emphasizes the critical need to translate these discoveries into precision medicine applications [68]. As a physiological stressor, pregnancy can expose latent cardiovascular vulnerabilities that may otherwise remain undetected. By acknowledging this and considering the impact of placental genetics and fetal sex on maternal vascular adaptation, healthcare providers can create more tailored and effective strategies for cardiovascular risk assessment, prevention, and management [69].

### 4.1. Sex-Based Screening and Risk Stratification

Women have historically been underrepresented in traditional cardiovascular risk assessments, which also rarely take sex-specific biology or reproductive history into consideration. Incorporating placental biomarkers, fetal sex, and pregnancy-related complications into cardiovascular risk models may enhance the early identification of women at elevated risk for hypertension and cardiovascular disease [70].

Integrating placental-derived circulating factors like soluble Flt-1, endoglin, and sex-specific microRNAs into clinical screening may allow for the early detection of endothelial dysfunction before overt hypertension develops [71]. Moreover, integrating maternal and fetal genetic differences into sex-specific polygenic risk scores may improve risk assessment and help pinpoint women who would benefit from early preventive care [72].

### 4.2. Personalized Prevention Strategies

Understanding the mechanisms underlying sex-specific hypertension risk enables the development of targeted prevention approaches. Women with hypertensive pregnancy histories, especially those linked to male fetuses or abnormal placental gene expression, may benefit from prioritized, individualized intervention approaches. This includes personalized lifestyle adjustments, ongoing blood pressure monitoring, and targeted pharmacological prophylaxis based on individual risk assessments [63,73].

Hormonal status also guides preventive therapy decisions; for instance, postmenopausal women or individuals with reduced estrogen signaling may benefit from treatments aimed at restoring vascular function or reducing OS. Furthermore, placental epigenetic markers that capture maternal–fetal interactions can help guide more precise approaches to nutrition and stress reduction [74].

### 4.3. Sex-Informed Therapeutic Management

The existing antihypertensive therapies have largely been developed and assessed without the consideration of sex-specific differences in drug metabolism, hormonal milieu, or underlying pathophysiological mechanisms [75]. The recognition of sex-specific molecular pathways in hypertension implies that therapeutics aimed at the renin–angiotensin system, OS, or inflammatory signaling may possess distinct efficacy and safety profiles in female patients [76].

Moreover, pregnancy history should inform pharmacologic decisions in women of reproductive age or during the perimenopausal transition, when vascular vulnerability is heightened. The discovery of sex- and placenta-specific molecular markers opens up promising possibilities for therapies, including epigenetic modulators and microRNA-based interventions targeting impaired vascular programming [77].

### 4.4. Toward Integrative Models and Longitudinal Care

Effective hypertension treatment requires a holistic, life-course approach that integrates both reproductive and cardiovascular health considerations. Predictive tools depend on electronic health records and biobanks, including detailed reproductive histories, fetal sex information, and genomic data. Multidisciplinary teams of obstetricians, cardiologists, and genetic counselors can collaborate to develop personalized monitoring and treatment plans [78].

Such approaches have the potential to reduce the disproportionate burden of hypertension and cardiovascular disease in women, advance maternal health outcomes, and personalize preventive treatments by accounting for sex-specific and pregnancy-related biological influences [79,80].

## 5. Future Directions

Despite substantial progress in uncovering sex differences in hypertension through placental genomics and pregnancy-related vascular programming, key research gaps persist that must be bridged to translate these insights into clinical application [69].

### 5.1. Need for Sex-Stratified and Longitudinal Studies

Although substantial progress has been made in understanding sex differences in hypertension through placental genetics and pregnancy-associated vascular programming, key research gaps must be addressed before these insights can be translated into clinical practice [81].

Previous research on hypertension and cardiovascular illness has historically underrepresented women or failed to stratify analyses by sex and reproductive factors [2]. Future research should prioritize explicit sex-stratified investigation into how sex chromosomes, hormones, and placental biology interact to influence vascular health across the lifespan. Longitudinal cohorts observing women before, during, and after pregnancy, combined with comprehensive fetal sex data, will be important in understanding temporal patterns of vascular remodeling and hypertension risk [69].

Furthermore, combining multi-omics approaches (genomics, epigenomics, transcriptomics, and proteomics) with clinical phenotyping can deepen mechanistic insights and identify biomarkers predictive of sex-based health outcomes. This research is pivotal to the advancement of precision medicine and to establishing its effectiveness in both sexes [82,83].

### 5.2. Enhancing Polygenic Risk Scoring with Sex and Pregnancy Context

Polygenic risk scores (PRSs) have emerged as powerful tools to quantify genetic susceptibility to hypertension. However, current PRS models are typically developed from mixed-sex cohorts and rarely incorporate pregnancy-related phenotypes or placental genomic data. Incorporating sex-specific genetic architectures, as well as maternal–fetal gene interactions, into PRS frameworks represents a promising frontier [84].

Developing sex- and pregnancy-informed PRSs could improve the early identification of women at risk for hypertensive disorders and guide preventive interventions. However, this will require large, diverse datasets with robust reproductive and fetal sex information, as well as standardized approaches to modeling gene–environment interactions [52].

### 5.3. Opportunities for Translational and Clinical Innovation

Emerging insights into sex differences in hypertension necessitate novel treatment approaches that target placental signaling networks, endothelial dysfunction, and epigenetic programming [85]. The development of sex-specific medicines, as well as biomarkers reflecting placental health and fetal sex, has the potential to improve the therapy of pregnancy-related hypertensive diseases and avoid the progression to chronic hypertension [45].

Interdisciplinary collaborations bridging obstetrics, cardiology, genomics, and bioinformatics will be essential to accelerate these translational efforts. Additionally, patient engagement and the inclusion of diverse populations in research will ensure that discoveries benefit all women globally [86].

## 6. Conclusions

Hypertension represents a quintessential example of a sex-differentiated disease, with distinct biological underpinnings shaped by genetic, hormonal, and placental factors. Pregnancy provides a distinctive and insightful framework for examining these differences, demonstrating how fetal sex and placental genomics regulate vascular programming in a way that affects both acute maternal cardiovascular adaptation and the long-term risk of hypertension.

Analyzing the molecular interaction between the placenta and maternal vasculature, focusing on sex-specific gene expression and epigenetic mechanisms, provides a greater understanding of fundamental biological processes and directs the advancement of precision medicine. Integrating sex-specific insights into risk assessment, preventive strategies, and therapeutic interventions enables clinicians to more accurately identify women at elevated risk and to adapt clinical approaches aimed at reducing hypertensive disorders and their long-term health effects.

These findings suggest a mechanistic paradigm in which placental-derived molecular mediators, including sFlt-1, sEng, HIFs, and sex-biased miRNAs, affect maternal angiogenic signaling, nitric oxide bioavailability, and endothelial integrity. These disturbances, particularly when combined with predisposed maternal genotypes (e.g., SH2B3, eNOS variations), form a pathophysiological link between hypertensive pregnancy problems and later-life cardiovascular disease. Understanding these interactions is critical for determining biological indicators and treatment targets in sex-specific vascular therapy.

Future research must prioritize sex-stratified studies, integrate multi-omics data, and refine polygenic risk models to fully capture the complexity of sex differences in hypertension. Translational efforts that bridge obstetrics and cardiovascular medicine hold great promise for reducing the global burden of hypertension among women.

Finally, using pregnancy as a natural cardiovascular stress test, combined with breakthroughs in placental genetics, may allow for a more individualized and egalitarian approach to hypertension therapy, hence enhancing cardiovascular health outcomes across the female lifespan.

## Figures and Tables

**Table 1:** Major epidemiological studies linking pregnancy complications to long-term hypertension.

Study/Cohort	Population Size	Key Finding	Relative Risk of Hypertension
Nurses’ Health Study	>600,000	PE linked to later HTN and CHD	~3.0
HUNT Study (Norway)	~15,000	Gestational HTN associated with future HTN	~2.4
UK Biobank	>100,000	HDP predictive of stroke, MI, HTN	2–4× increase
CHAMPS Study (Canada)	~20,000	Risk persists up to 20 years postpartum	Sustained elevation

**Table 2:** Key placental genes and pathways involved in maternal cardiovascular regulation.

Gene/Pathway	Function	Alteration in Hypertensive Pregnancy	Impact on Maternal Vasculature
*FLT1* (sFlt-1)	Anti-angiogenic factor, VEGF inhibitor	Elevated	Endothelial dysfunction, hypertension
*ENG* (sEng)	TGF-β antagonist, modulates vascular integrity	Elevated	Vascular stiffness, inflammation
*VEGFA*, *PGF*	Pro-angiogenic factors	Downregulated	Impaired vascular remodeling
*HIF1A*, *HIF2A*	Hypoxia response regulators	Upregulated in placental hypoxia	Poor trophoblast invasion, systemic vasoconstriction

## Data Availability

No new data were created or analyzed in this study. Data sharing is not applicable to this article.
